# Nimodipine as Vasodilator in Guide Catheter Flush to Prevent Vasospasm During Endovascular Stroke Treatment

**DOI:** 10.1007/s00062-024-01424-0

**Published:** 2024-05-31

**Authors:** Louisa J. Sommer, Jessica Jesser, Omid Nikoubashman, Thanh N. Nguyen, Joao Pinho, Arno Reich, Martin Wiesmann, Charlotte S. Weyland

**Affiliations:** 1 Department of Neuroradiology, Aachen University Hospital, Aachen, Germany; 2 Department of Neuroradiology, Heidelberg University Hospital, Heidelberg, Germany; 3 Interventional Neurology and Neuroradiology, Boston Medical Center, Boston, MA USA; 4 Department of Neurology, Aachen University Hospital, Aachen, Germany; 5 Department of Diagnostic and Interventional Radiology, University Hospital Bonn, Venusberg-Campus 1, 53127 Bonn, Germany

**Keywords:** Mechanical thrombectomy, Complication, Vasospasm, Calcium channel blocker, Nimodipine

## Abstract

**Purpose:**

The clinical importance and management of vasospasm as a complication during endovascular stroke treatment (EVT) has not been well studied. We sought to investigate the effect of adding nimodipine to the guiding catheter flush (GCF) to prevent vasospasm during EVT.

**Methods:**

This is a single-center retrospective analysis including patients with EVT (stent-retriever and/or distal aspiration) treated for anterior or posterior circulation intracranial vessel occlusion from January 2018 to June 2023. Exclusion criteria were intracranial or extracranial stenosis, intra-arterial alteplase, patient age over 80 years. Study groups were patients with (nimo+) and without (nimo−) nimodipine in the GCF. They were compared for occurrence of vasospasm as primary endpoint and clinical outcome in univariate analysis.

**Results:**

477 patients were included in the analysis (nimo+ *n* = 94 vs. nimo− *n* = 383). Nimo+ patients experienced less vasospasm during EVT (e.g. vasospasm in target vessel *n* (%): nimo− = 113 (29.6) vs. nimo+ = 9 (9.6), *p* < 0.001; extracranial vasospasm, *n* (%): nimo− = 68 (17.8) vs. nimo+ = 7 (7.4), *p* = 0.017). Patients of the two study groups had a comparable clinical outcome (90 day mRS, median (IQR): 3 (1–6) for both groups, *p* = 0.896). In general, patients with anterior circulation target vessel occlusion (TVO) experienced more vasospasm (anterior circ. TVO 38.7% vs. posterior circ. 7.5%, *p* = 0.006).

**Conclusion:**

Prophylactic adding of nimodipine reduces the risk of vasospasm during EVT without affecting the clinical outcome. Patients with anterior circulation TVO experienced more vasospasm compared to posterior circulation TVO.

## Introduction

More than 10% of endovascular stroke treatments (EVTs) for acute ischemic stroke are associated with periprocedural complications [1]. Iatrogenic vasospasm is a common complication (3.9–23%) that occurs due to vessel wall irritation during device instrumentation or thrombectomy retrieval maneuvers [2–4]. Extracranial vasospasm of the parent vessel arteries occur when placing guide catheters and intracranial vasospasm occurs during stent retriever or contact aspiration placement. On angiography, vasospasm can be perceived as a concentric contraction of the arterial vessel wall.

Vasospasm is more likely to occur in younger stroke patients as well as in patients who undergo EVT with multiple thrombectomy attempts [5]. While vasospasm during EVT is considered as non-serious and a transient complication by many neurointerventionalists [6], others discuss vasospasm as a potential cause for extending infarction after EVT and associations with further complications in subsequent EVT maneuvers, including “snagging” of stent-retrievers, or stroke recurrence [7]. The clinical relevance of vasospasm as a complication during EVT remains uncertain as well as geographical differences in incidence rates or related clinical sequelae.

Intra-arterial application of vasodilators, such as calcium channel blockers (CCBs), can resolve vasospasm in most cases [8]. CCBs can be added to catheter flushes to prevent vasospasm during EVT or they can be given as an intra-arterial bolus via the intermediate or guide catheter after detection of vasospasm. There are currently no guidelines available for the treatment of vasospasm and local treatment protocols vary [9]. Adding vasodilators prophylactically to guide catheter flushes (GCF) to prevent vasospasm is left to the discretion of the neurointerventionalists. We aimed to evaluate the effect of adding nimodipine as vasodilator to the GCF on the occurrence of vasospasm during EVT and on the clinical outcome after acute stroke and EVT.

## Methods

**Fig. 1:**
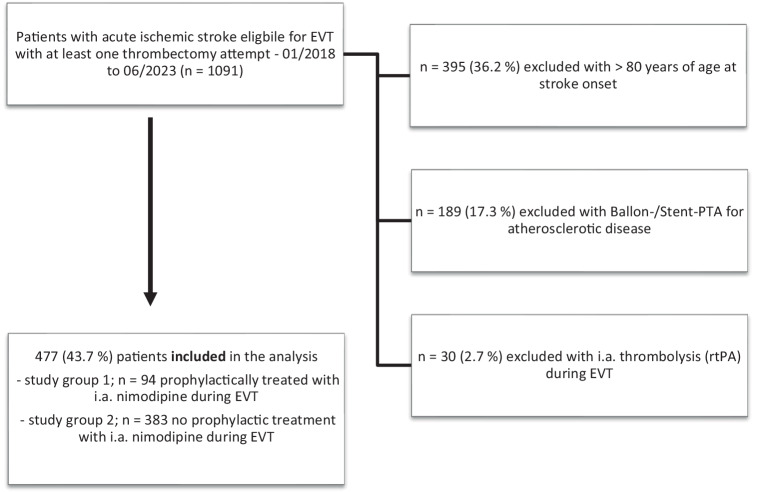
Patient selection and study-groups for analysis—patient selection for comparison of patients with intra-arterial nimodipine started before endovascular stroke treatment (*EVT*) with the guiding catheter flush for anterior and posterior circulation acute ischemic stroke vs. no intra-arterial nimodipine during EVT. (*PTA* percutaneous transluminal angioplasty, *rtPA* recombinend-tissue Plasminogen Activator, *EVT* endovascular stroke treatment)

This was a single-center retrospective analysis based on the local stroke databank of a tertiary german stroke care center (University Hospital RWTH Aachen). This study was performed in line with the principles of the Declaration of Helsinki. Ethics approval was granted by the local ethics committee. Written informed consent was waived for this analysis in view of the retrospective nature of the study and all the procedures performed were part of the routine care. The study was conducted according to the STROBE criteria [10]. Patients with intracranial vessel occlusion of the anterior or the posterior circulation consecutively treated with EVT between January 2018 and June 2023 were included in the analysis. Exclusion criteria were patient age over 80 years at stroke onset, intracranial or extracranial stenosis leading to balloon-assisted percutaneous transluminal angioplasty (PTA) and/or stent-angioplasty and intra-arterial thrombolysis (with rt-PTA) during EVT (Fig. 1).

Study groups were formed according to the adjunctive application of nimodipine as vasodilator in the guide catheter flush (GCF).

### Local Standard of EVT

At the study site, EVT was performed exclusively by trained neurointerventionalists. In summary, the thrombectomy technique is at the neurointerventionalists’ discretion and performed mostly with a stent-retriever with or without an additional distal aspiration catheter (SAVE or Solumbra technique [6, 11, 12]) or distal aspiration alone. For anterior and posterior circulation intracranial vessel occlusions, the standard access route during the study period was via the groin. After placing a short introducer sheath (8 F or 9 F), a guide catheter is placed in the internal carotid artery leading to the target vessel occlusion (mostly 8 F FlowGate, Stryker, Kalamazoo, Michigan, USA) or in a vertebral artery in case of posterior circulation target vessel occlusion (mostly 8 F Cordis, Miami Lake, Florida, USA or 8 F AXS Infinity Plus, Stryker, Kalamazoo, Michigan, USA). Further intracranial access is usually performed with a triaxial system involving an intermediate aspiration catheter (mostly AXS Catalyst 5 F or 6 F, Stryker or SOFIA 5 F, MicroVention, Aliso Viejo, California, USA) and a microcatheter (mostly Rebar18, Medtronic, Dublin, Ireland or Trevo Trak 21, Stryker, Kalamzoo, Michigan, USA). Thereafter, the intracranial target vessel occlusion is traversed in case of primary stent retriever maneuver or the aspiration catheter is placed at the proximal aspect of the occlusion for primary aspiration maneuver. All catheters are constantly flushed with heparinized flushing with a flow volume of approximately 3–4 mL/h. The saline bags for continuously flushing the catheters in use were prepared with 1000 international units of heparin. There is no local protocol advocating for or against the use of nimodipine in the guide catheter flush. Adding 10–20 ml nimotop (2–4 mg nimodipine, respectively) (Bayer Vital, Germany) as vasodilator to the guide catheter flush is left to the discretion of the treating neurointerventionalist. All drugs administered during EVT are documented in an obligatory intervention protocol by the interventionalist in charge and by the technical assistant in a second document.

### Study Endpoints

The primary study endpoint was the occurrence of vasospasm during EVT. Secondary endpoint of the study is the clinical outcome according to mRS (modified Rankin Scale) 90 days after stroke onset. Severe vasospasm was defined as concentric reduction of vessel lumen by more than 50% without signs of remaining thrombus or atherosclerotic stenosis using DSA (digital-subtraction angiography) [13, 14]. Angiograms and intervention protocols were reviewed by two readers blinded for nimodipine administration, one radiologist in training (2 years of experience) and one neurointerventionalist (7 years of experience). Consensus reading was performed in case of inter-reader disagreement involving a third interventional neuroradiologist (14 years of experience).

### Statistical Analysis

Data are shown as number of events and percentage (*n*, %) and median with interquartile range (IQR) after testing for normal distribution with the Kolmogorow-Smirnow test or Shapiro-Wilk test. Further analysis was conducted with the Mann-Whitney-U-Test or χ2 test and Fisher’s exact test to compare groups, as appropriate. All tests were two-sided and a *p*-value of < 0.05 considered statistically significant. Multiple testing was corrected with Benjamini-Hochberg corrections. Statistical analyses were performed with SPSS Statistics (29.0; IBM, Armonk, NY). In cases of Missing values, we adjusted the denominator and delineated these adjustments in the footnotes accompanying the tables.

### Funding and Declarations Statement

This research was not funded by external sources. The authors did not receive any support from any organization for the submitted work. The authors have no relevant financial or non-financial interest to disclose.

## Results

Among 477 patients receiving EVT with at least one thrombectomy attempt between 01/2018 and 06/2023, nimodipine was added to the guide catheter flush before the beginning of the intervention (nimo+ study group) in 94 (19.7%) patients. This cohort was compared with 383 (80.3%) patients who did not receive prophylactic nimodipine or other vasodilator in the guide catheter flush (nimo− control group). Overall, the median (interquartile range, IQR) age was 69 (60–76), and 217 (45.5%) were female. There was no difference in the groups with regards to baseline characteristics, vascular risk factors, age, sex or relevant stroke-related co-morbidities—Table 1.

**Table 1:** Group comparison according to nimodipine in guide catheter flush for endovascular stroke treatment

	Nimo− (*n* = 383)	Nimo+ (*n* = 94)	*p*-value
*Patient Characteristics*			
Age [years], median (IQR)	69 (60–76)	67 (69–75)	0.439^^^
Male, *n* (%)	203 (53.0)	57 (60.6)	0.183^*^
Hypercholesterolemia, *n* (%)	102 (30.1) ^mv^ ^=^ ^44^	30 (35.3) ^mv^ ^=^ ^9^	0.294^*^
Atrial fibrillation, *n* (%)	147 (42.5) ^mv^ ^=^ ^37^	32 (37.2) ^mv^ ^=^ ^8^	0.505^*^
Hypertension, *n* (%)	271 (75.9) ^mv^ ^=^ ^26^	68 (78.2) ^mv^ ^=^ ^7^	0.816^*^
Type 2 diabetes mellitus, *n* (%)	92 (26.8) ^mv^ ^=^ ^40^	24 (27.6) ^mv^ ^=^ ^7^	0.837^*^
Smoker, *n* (%)	97 (28.3) ^mv^ ^=^ ^40^	22 (25.9) ^mv^ ^=^ ^9^	0.235^*^
Alcohol abuse, *n* (%)	18 (5.4) ^mv^ ^=^ ^49^	7 (8.4) ^mv^ ^=^ ^11^	0.152^*^
*Medication before index stroke*			
Oral Anticoagulation (including DOAC), *n* (%)	82 (22.8) ^mv^ ^=^ ^24^	21 (23.6) ^mv^ ^=^ ^5^	0.483^*^
Antiplatelet medication ASA *n* (%)	70 (19.6) ^mv^ ^=^ ^25^	13 (14.9) ^mv^ ^=^ ^7^	0.376^*^
Antiplatelet medication Clopidogrel, *n* (%)	9 (2.5) ^mv^ ^=^ ^28^	3 (3.4) ^mv^ ^=^ ^7^	0.390^*^
*Stroke related clinical, imaging and outcome parameters*			
Pre-stroke mRS, median (IQR)	0 (0–0) ^mv^ ^=^ ^60^	0 (0–0) ^mv^ ^=^ ^11^	0.159^^^
Admission mRS, median (IQR)	4 (3–4) ^mv^ ^=^ ^34^	4 (3–4) ^mv^ ^=^ ^11^	0.177^^^
90 day follow-up mRS, median (IQR)	3 (1–6) ^mv^ ^=^ ^123^	3 (1–6) ^mv^ ^=^ ^24^	0.896^^^
NIHSS score baseline, median (IQR)	13 (8–18) ^mv^ ^=^ ^14^	14 (6–18) ^mv^ ^=^ ^4^	0.694^^^
*Location of target vessel occlusion*			
MCA M1 segment, *n* (%)	138 (36)	35 (37.2)	0.828^*^
MCA M2 segment, *n* (%)	92 (24.1)	21 (22.3)	0.844^*^
MCA ≥ M3 segment, *n* (%)	8 (2.1)	0	0.368^*^
ACA, *n* (%)	16 (4.2)	2 (2.1)	0.35^*^/0.547^+^
ICA distal, *n* (%)	14 (3.7)	0	0.06^*^/0.083^+^
Carotid T, *n* (%)	57 (14.9)	20 (21.3)	0.131^*^
Vertebral artery, *n* (%)	7 (1.8)	1 (1.1)	0.605^*^
Basilar artery, *n* (%)	31 (8.1)	3 (3.2)	0.098^*^
PCA, *n* (%)	11 (2.9)	2 (2.1)	0.651^*^
*Procedure times*			
Onset to Image, median (IQR)	135 (78.5–215.8) ^mv^ ^=^ ^147^	108 (72.8–230.8) ^mv^ ^=^ ^44^	0.683^^^
Image to EVT start, minutes, median (IQR)	48 (37–66) ^mv^ ^=^ ^47^	59 (47.3–82.8) ^mv^ ^=^ ^14^	**<** **0.001**^**^**^
EVT start to final recanalization, median (IQR)	45 (30–83) ^mv^ ^=^ ^38^	52 (35.8–68.8) ^mv^ ^=^ ^10^	0.233^^^
*Procedural aspects*			
Number of thrombectomy attempts, median (IQR)	1 (1–3)	2 (1–3)	0.288^^^
Number of stent retriever maneuvers, median (IQR)	1 (1–2)	2 (1–3)	0.081^^^
Number of aspiration maneuvers, median (IQR)	0 (0–0)	0 (0–0)	0.101^^^
General anesthesia for EVT, *n* (%)	312 (89.1) ^mv^ ^=^ ^33^	70 (82.4) ^mv^ ^=^ ^9^	0.125^*^
*Post-procedural intracranial bleeding*			
ICH, *n* (%)	70 (18.3)	13 (12.2)	0.303^*^
SAH, *n* (%)	16 (4.9) ^mv^ ^=^ ^56^	3 (3.4) ^mv^ ^=^ ^7^	0.567^*^
Hemorrhagic transformation, *n* (%)	60 (18.4) ^mv^ ^=^ ^56^	12 (13.8) ^mv^ ^=^ ^7^	0.319^*^
SDH, *n* (%)	1 (0.3) ^mv^ ^=^ ^56^	0 (0) ^mv^ ^=^ ^7^	0.606^*^
*mTICI after treatment*			
0, *n* (%)	11 (2.9)	5 (5.3)	0.428^*^
1, *n* (%)	6 (1.6)	0	0.429^*^
2a, *n* (%)	10 (2.6)	2 (2.1)	0.862^*^
2b, *n* (%)	67 (17.5)	18 (19.1)	0.805^*^
2c, *n* (%)	24 (6.3)	7 (7.4)	0.802^*^
3, *n* (%)	237 (61.9)	54 (57.4)	0.714^*^

Bold values are statistically significant *p*-values (< 0.05)

*nimo+* nimodipine was added to the flush, *nimo−* no vasodilator in catheter flush during EVT, *mRS* modified Rankin Scale, *NIHSS* National Institutes Health Stroke Score, *ASPECTS* Alberta Stroke Programm Early CT Score, *mTICI* modified treatment in cerebral infarction, *MCA* middle cerebral artery, *ACA* anterior cerebral artery, *ICA* internal carotid artery, *PCA* posterior cerebral artery, *EVT* endovascular stroke treatment, *ICH* intracerebral hemorrhage, *SAH* subarachnoid hemorrhage, *SDH* subdural hematoma, *IQR* interquartile range, *mv* missing values due to lack of collected data

^*^Chi-Square-Test

^^^Mann-Whitney-U-Test

^+^Fisher exact test

For the primary endpoint of the occurrence of vasospasm, the nimo+ study group had a lower incidence of intracranial vasospasm during EVT (9/94 (9.6%) vs. 113/383 (29.6%), *p* < 0.001). Extracranial and intracranial vasospasm was observed less frequently in the nimo+ study group (Table 2).

**Table 2:** Group comparison of patients with prophylactic vasodilator in catheter flush during endovascular stroke treatment regarding the development of vasospasm during the procedure

	Nimo− *N* = 383	Nimo+ *N* = 94	*p*-value
Any vasospasm during intervention, *n* (%)	113 (29.5) ^mv^ ^=^ ^1^	9 (9.6)	**<** 0.001^*^
Vasospasm in extracranial artery, *n* (%)	68 (17.8) ^mv^ ^=^ ^2^	7 (7.4) ^mv^ ^=^ ^3^	0.017^*^
Intracranial vasospasm in target vessel, *n* (%)	62 (16.2) ^mv^ ^=^ ^2^	3 (3.2) ^mv^ ^=^ ^1^	**<** 0.001^*^

Bold values are statistically significant *p*-values (< 0.05)

*mv* missing values due to missing imaging of vessels during endovascular stroke treatment

^*^Chi-Square-Test

^^^Mann-Whitney-U-Test

^+^Fisher exact test

With regards to the procedure times, imaging to EVT start time was longer in the nimo+ study group (median (IQR): 59 min (47.3–82.8) in the nimo+ study group vs. 48 min (37–66) in the nimo− control group, *p* < 0.001), whereas other procedural times did not differ significantly (Table 1).

Independent of nimodipine in the GCF, we observed a higher rate of vasospasm in patients with anterior circulation target vessel occlusion—Table 3.

**Table 3:** Group comparison of patients with anterior and posterior circulation target vessel occlusion regarding the development of vasospasm during endovascular stroke treatment

	Anterior circulation target vessel occlusion *N* = 417	Posterior circulation target vessel occlusion *N* = 61	*p*-value
Incidence of any vasospasm during EVT, *n* (%)	118 (38.7) ^mv^ ^=^ ^77^	4 (7.5)	0.006^*^
Intracranial vasospasm, *n* (%)	56 (13.4)	6 (9.8)	**<** 0.001^*^
Extracranial vasospasm, *n* (%)	72 (17.3)	0 (0)	**<** 0.001^*^

Bold values are statistically significant *p*-values (< 0.05)

*EVT* endovascular stroke treatment, *mv* missing values due to missing imaging of vessels during endovascular stroke treatment

^*^Chi-Square-Test

^^^Mann-Whitney-U-Test

^+^Fisher exact test

In the context of EVT, the number of thrombectomy attempts was comparable between study groups. For both groups, a greater number of attempts was performed utilizing stent retriever as opposed to aspiration maneuvers (stent retriever maneuvers, median (IQR): nimo− 1 (1–2) vs. nimo+ 2 (1–3), *p* = 0.081 vs. aspiration maneuvers, median (IQR): 0 (0–0) for both groups, *p* = 0.101).

Regarding the possibility of post-procedural risks such as intracranial hemorrhage, the two study groups did not vary (for example, incidence of intracerebral hemorrhage (ICH), *n* (%): nimo− 70 (18.3) vs. nimo+ 13 (12.2), *p* = 0.303, see Table 1). As for the secondary study endpoint, the patient outcomes did not differ between the two study groups (90 day post stroke mRS, median (IQR): 3 (1–6) for both study groups, *p* = 0.896).

## Discussion

Vasospasm is a common complication during endovascular stroke treatment (EVT) [4]. With this study, we demonstrated that patients, prophylactically treated with nimodipine as vasodilator via the guide catheter flush (GCF), might experience vasospasm less often during EVT. Also, we found patients treated for anterior circulation target vessel occlusion to be more likely to experience vasospasm during EVT compared to posterior circulation target vessel occlusion independent of prophylactic treatment with nimodipine. At nearly 30%, the general rate of vasospasm is high in this study compared to the literature [1–4]. This is probably due to the younger study cohort (patients > 80 years of age are excluded), the varying definition of vasospasm (> 50% concentric stenosis in this study) and a probable underrepresentation in the literature (non-relevant vasospasms might not be reported continuously by neurointerventionalists).

Presently, there is uncertainty regarding whether vasospasm, as complication during EVT, manifests transiently and as asymptomatic complication, or has a negative effect on patient outcome. Recent studies suggest that vasospasm might influence patient outcome for EVT with target vessel occlusions of the anterior circulation. For posterior circulation target vessel occlusions we found that vasospasm is less likely to occur. For both, anterior and posterior circulation target vessel occlusion, the clinical outcome, represented by the 90 d mRS, was not affected by vasospasm, independent of the prophylactic administration of nimodipine.

### Limitations

This study is limited by the mono-centric retrospective survey design. There is also no standard operation procedure regarding the administration of vasodilators to the guide catheter flush, so that inter-individual differences regarding neurointerventionalists might influence the study results. We also did not analyze the exact thrombectomy maneuver technique (e.g. stent retriever assisted thrombus extraction, e.g. SAVE vs. SOLUMBRA technique, or thrombus aspiration). Differing techniques might be connected to differing risks of causing vasospasm.

Another limitation of this study pertains to the comparatively small patient cohort receiving prophylactic nimodipine treatment (nimo+ group 94 patients vs. nimo− group 383 patients).

Up until now, there is no standardized approach at our clinic regarding the prophylactic treatment with nimodipine; consequently, the decision was left to each interventionalist.

## Conclusion

In this study, adding nimodipine to the guide catheter flush reduced the occurrence rate of vasospasm during endovascular stroke treatment for anterior and posterior circulation target vessel occlusions. Moreover, vasospasm was found to be a complication predominantly associated with anterior circulation target vessel occlusions as compared to posterior circulation target vessel occlusion.
